# Microbial diversity and functional genes of red vinasse acid based on metagenome analysis

**DOI:** 10.3389/fmicb.2022.1025886

**Published:** 2022-10-13

**Authors:** Jianman Lv, Yaolu Ye, Yuan Zhong, Wukang Liu, Meilin Chen, Ailing Guo, Jun Lv, Huawei Ma

**Affiliations:** ^1^College of Food Science and Technology, Huazhong Agricultural University, Wuhan, China; ^2^Institute of Infection and Immunity, Taihe Hospital, Hubei University of Medicine, Shiyan, China; ^3^Aquatic Preservation and Processing Technology, Guangxi Academy of Fishery Science, Nanning, China

**Keywords:** red vinasse acid, metagenome, microbial diversity, glucose metabolism, functional genes, amino acid

## Abstract

Red vinasse acid has a distinct flavor and a vivid red color that are directly tied to the intricate metabolic activities of microorganisms that produce it. In this study, metagenomic technology was used to mine its functional genes and examine the microbial diversity of red vinasse acid. The findings revealed the identification of 2,609 species, 782 genera, and 63 phyla of microorganisms, and the dominant genus was *Lactobacillus*. Amino acid metabolism and carbohydrate metabolism were significant activities among the 16,093 and 49,652 genes that were annotated in the evolutionary genealogy of genes: Non-supervised Orthologous Groups (eggNOG) and Kyoto Encyclopedia of Genes and Genomes (KEGG) databases, respectively. In gluconeogenesis, red vinasse acid encodes 194 genes controlling the transporter protein systems of different sugars and has key enzyme genes that catalyze the conversion of intracellular sugars into glycolytic intermediates. In amino acid flavor formation, red vinasse acid contains 32 control genes for branched-chain aminotransferase (BCAT), 27 control genes for aromatic-amino-acid transaminase (ArAT), 60 control genes for keto acid invertase, 123 control genes for alcohol/aldehyde dehydrogenase, and 27 control genes for acetyl esterase, which have the basis for the formation of strong flavor substances from amino acids.

## Introduction

Red vinasse acid (Bai et al., 2020) is a kind of unique flavor food in Guangxi, China. It is a well-known regional specialty that is mostly produced in Guangxi Wuxuan and Guangxi Guiping. Bright crimson, crisp., and delectable red vinasse acid is pickled with red rice as seeds. Red yeast rice was fermented at a specified temperature and duration under the combined influence of *Monascus*, *Lactobacillus*, *Yeast*, and other microorganisms to create red vinasse acid (Bai et al., 2020). Its diverse microbial community, which includes the dominant fungus *Monascus* and the dominant bacteria *Lactobacillus*, is strongly associated with the development of red vinasse acid’s distinctive flavor and functional features. Numerous secondary metabolites, such as *Monascus* pigments (MPs), monacolin K, γ-aminobutyric acid (GABA), and dimerumic acid, can be produced by *Monascus* (Aniya et al., 2000; Diana et al., 2014; Chen et al., 2015). In addition to effectively lowering blood pressure, lowering plasma cholesterol, preventing obesity, improving anti-inflammatory activity, preventing cancer, lowering blood sugar levels, and having anti-inflammatory and anti-tumor properties, MPs can replace some synthetic pigments in the food industry (Nam et al., 2014; Zhang et al., 2021). The most important effect of GABA is blood pressure regulation. Numerous studies have shown that GABA can reduce high blood pressure in animals and humans (Yoshimura et al., 2010; Yang et al., 2012; Pouliot-Mathieu et al., 2013; Tsai et al., 2013). Monacolin K can block cholesterol synthesis pathways, reduce cholesterol synthesis, and thus play a role in lowering blood lipids (Hsieh et al., 2013; Ajdari et al., 2014; Dikshit and Tallapragada, 2015, 2016). Lactobacillus has a variety of probiotic functions, such as promoting the absorption of nutrients, improving human immunity, and maintaining the balance of intestinal flora (Arnold et al., 2018; Riaz Rajoka et al., 2018; Sandes et al., 2018; Chen et al., 2020). Therefore, Red vinasse acid thus plays a significant role in healthcare. In recent years, it has been possible to estimate the microbiological diversity of red vinasse acid detected by 16srRNA technology only at the genus level and not with sufficient accuracy at the species level. The discovery of functional genes has some restrictions.

New insights into the roles played by natural microbial communities have been made possible by the introduction of metagenomic methods (Knight et al., 2018). As a result, the integration of the genetic, taxonomic, and functional diversity of the microbial community has drawn more and more attention. A study technique called metagenomics uses gene sequences to analyze the microbial data contained in environmental samples (Mori et al., 2008; Xie et al., 2020). It has developed into a crucial tool for researching microbial diversity and community traits because of its ability to swiftly and reliably gather a wealth of biological data and extensive microbiological information. A metagenomic technique, as opposed to 16srRNA high-throughput sequencing, can not only fully mine the microbial genome information but also study the function and metabolic pathways. Based on this information, it is more convenient to mine the biodiversity, community structure, functional characteristics, and relationships of microflora (Li et al., 2017; Belleggia et al., 2020). Importantly, when compared to conventional rRNA gene sequencing, metagenomic sequencing can identify novel species with greater resolution and accuracy (Chen et al., 2017). Our understanding of the microbial communities in fermented foods has significantly improved over the past several decades as a result of advancements in next-generation DNA sequencing and high-throughput computational methods (Arıkan et al., 2020). Studies on wines (Johansson et al., 2021), kimchi (Kim et al., 2021), fermented Chinese xiaoqu (Su et al., 2020), dajiang-meju (Xie et al., 2019), and Turkish kefir grains (Ilıkkan and Bağdat, 2021) are a few examples.

Studies on the microbiological diversity of red vinasse acid have been conducted recently. Further study is necessary since it is unclear what species of microorganisms exist and because functional genes are nearly at the blank stage. In order to provide a theoretical foundation for the development of the red vinasse acid industry and the development of Monascus resources, metagenomics, and bioinformatics analysis methods were used in this study to identify the core microbial groups at the ‘species’ level of taxonomic status, analyze the microbial diversity of red vinasse acid, and excavate functional genes in group microorganisms.

## Materials and methods

### Experimental materials

#### Composition and production process of red vinasse acid

Fresh edible fruits and vegetables (or edible fruit and vegetable salted billet), red vinasse as the main raw material, then add edible salt, add or not add sugar (sugar or rock sugar, etc.), rice-flavored white wine, garlic, chili pepper, star anise, and other auxiliary ingredients, through the raw and auxiliary materials pretreatment, cleaning, drying, mixing, vinasse fermentation, packaging, and other processes made ready to eat red vinasse acid (Bai et al., 2020).

#### Source and storage of experimental samples

The samples of red vinasse acid were collected from Guangxi in June, 2021, and then brought back to the laboratory and sent to Biomarker Technologies for metagenome sequencing immediately, and the remaining samples were stored in the laboratory refrigerator at 4°C.

### Total DNA extraction

Take about 500 mg of red vinasse acid tissue sample into a domestic 2 ml centrifuge tube, add pre-cooled steel beads, place the taken tissue sample in liquid nitrogen, and grind it thoroughly at 1,500 rpm for DNA extraction.

Complete the nucleic acid extraction using the CTAB procedure, measure the concentration of the extracted nucleic acid using the Qbiut (Invitrogen, model Qubit3.0, reagent Qubit TM dsDNA HS Assay Kit), and assess the integrity using 1% agarose gel electrophoresis. Red vinasse acid sample processing by Biomarker Technologies included DNA extraction and metagenome sequencing.

### Metagenome library construction and sequencing methods

#### Library construction methods

##### Genomic enzymatic disruption and end repair

1. Take 10 ng of genomic DNA (Qubit quantification) into a 96-well plate, make up to the corresponding volume with Unclease-free Water, finally add the corresponding digestion reagent (on ice), mix well by pipetting, centrifuge instantly, and perform the reaction immediately in a PCR instrument (Eppendorf).
2. Rapid Ligation Butter 3, ligase and index are added to the digestion product, blown, and mixed, centrifuged instantaneously, and placed in the PCR instrument for reaction.

##### Conjugate product purification

1. Add a certain volume of magnetic beads to the conjugate product, mix thoroughly, incubate at room temperature, and place on a magnetic stand, let stand, and carefully remove the supernatant.
2. Add freshly prepared 80% ethanol to the tube, let it stand on the magnetic rack for 30s, and remove the supernatant.
3. Repeat Step 2 once, after the second wash, centrifuge instantaneously and remove the supernatant with a 10 μl gun.
4. Place the reaction tube on the magnetic rack, open the cap and air dry until the surface of the magnetic beads is lusterless and has fine cracks.
5. Remove the reaction tube from the magnetic rack, add Unclease-free Water to elute DNA, blow and mix well, incubate at room temperature, and then rest on the magnetic rack, carefully aspirate the supernatant into a new PCR tube.

##### PCR amplification

1. Add various reagents required for PCR reaction, mix well, and centrifuge.
2. According to the PCR reaction conditions, put it into the PCR instrument for PCR amplification.

##### PCR product fragment screening

1. Instantaneously centrifuge the PCR reaction tube, add the corresponding volume (0.6×) of magnetic beads, and mix thoroughly.
2. Incubate at room temperature, then place on a magnetic stand, and carefully transfer the supernatant to a new PCR tube.
3. Aspirate the corresponding volume (0.12×) of beads into the supernatant of Step 2, gently blow and mix well, incubate at room temperature, then place on a magnetic stand, let stand, carefully remove the supernatant.
4. Add freshly prepared 80% ethanol to the reaction tube, let it stand on the magnetic rack for 30s, remove the supernatant, and repeat the Step 1 time.
5. Open the cap of the tube, air dry until the surface of the magnetic beads is lusterless with fine cracks, add Unclease-free Water to elute the library, blow and mix, let stand at room temperature, let stand on the magnetic stand, carefully aspirate the supernatant into a new 1.5 ml centrifuge tube, and store at −20°C for backup.

##### Library quality control

The library was quality-checked by Qsep-400 and the library concentration was quantified using Qubit 3.0, and meets the following indicators can be tested on the machine: Concentration ≥ 1 ng/μl, center value of fragment 430–530 bp, average value between 420 and 580 bp, normal distribution of peak pattern, single fragment without spurious peak.

#### Sequencing methods

The constructed libraries were sequenced using illumina novaseq6000 (San Diego, CA, United States). The kit NovaSeq 6,000 S4 Reagent Kit (San Diego, CA, United States) was used for sequencing.

### Data analysis

#### Sequencing data quality control

The raw sequences (Raw reads) obtained from sequencing contain low quality sequences. To ensure the quality of information analysis, raw reads need to be filtered to obtain clean reads for subsequent information analysis. The main steps of data filtering are as follows.

1. Filtering raw tags to obtain high quality sequencing data (clean tags) using the software Trimmomatic (version v0.33 parameters PE LEADING:3 TRAILING:3 SLIDINGWINDOW:50:20 MINLEN:120).
2. Use bowtie2 (version v2.2.4 parameter—seed 123,456-I 200-X 1000—un-conc) to compare with host genome sequence to remove host contamination (if a host reference genome is provided).

#### Metagenome assembly

Metagenome assembly was performed using the software MEGAHIT (Li et al., 2015; version v1.1.2 default parameters), filtering contig sequences shorter than 300 bp. The assembly results were evaluated using QUAST (Gurevich et al., 2013; version v2.3 default parameters) software.

#### Metagenome component analysis

##### Gene prediction

MetaGeneMark (Zhu et al., 2010; version v.3.26 default parameters) software (http://exon.gatech.edu/meta_gmhmmp.cgi, Version 3.26) was used to identify coding regions in the genome using default parameters (parameters -A -D -f G).

#### Construction of non-redundant gene sets

Redundancy was removed using MMseq2 software (https://github.com/soedinglab/mmseqs2, Version 11-e1a1c) with the similarity threshold set to 95% and the coverage threshold set to 90%.

#### Functional annotation analysis

##### eggNOG functional annotation

Evolutionary genealogy of genes: Non-supervised Orthologous Groups (eggNOG; Powell et al., 2014; evolutionary genealogy of genes: Non-supervised Orthologous Groups) is a database of biological direct homologous gene clusters, which is a continuous update of the COG database. For specific annotation, the protein sequences of non-redundant genes are compared with the eggNOG database by BLAST (diamond v0.9.29 matching threshold *E*-value 1e−5) to find the most similar sequence in the eggNOG database, and the annotation and classification information corresponding to this sequence is the annotation and classification information of the corresponding sequenced genomic genes.

##### KEGG functional annotation

Kyoto Encyclopedia of Genes and Genomes (KEGG; Kanehisa et al., 2004) is a database for collecting genomes, biological pathways, diseases, drugs, and chemicals. For specific annotation, the protein sequences of non-redundant genes are compared with those included in the KEGG database by BLAST (diamond v0.9.29 matching threshold *E*-value 1e−5) to find the most similar sequence in the KEGG database, and the annotation information, corresponding KO number, and position in the KO corresponding pathway of the sequence is the annotation information of the corresponding gene in the sequenced genome. The annotation information, KO number and position in the biological process pathway of the corresponding gene in the KEGG database is the sequence with KO sequence number.

##### CAZy function annotation

Carbohydrate-active enzymes database (CAZy; Cantarel et al., 2009) is a database of carbohydrate-active enzymes, formed by the collection and classification of published literature and related proteins, and carefully maintained by experts. For the specific annotation, the hmmer (version 3.0) software was used to compare the protein sequences of the non-redundant genes separately with the Hidden Markov Model of each family of the CAZy database (matching parameters default, default screening threshold “if alignment >80aa, use *E*-value <1e−5, otherwise use *E*-value <1e−3; covered fraction of HMM > 0.3”) to find all families that satisfy the filtering threshold, so that the carbohydrate-active enzymes in the genome can be identified and analyzed for the presence of several conserved carbohydrate-related functional domains.

#### Species annotation and statistics

Based on the species information of the above non-redundant genes compared to the sequences in Nr, the species composition and relative abundance information of the samples were obtained.

## Results

### Microbial diversity analysis of red vinasse acid

**Figure 1:**
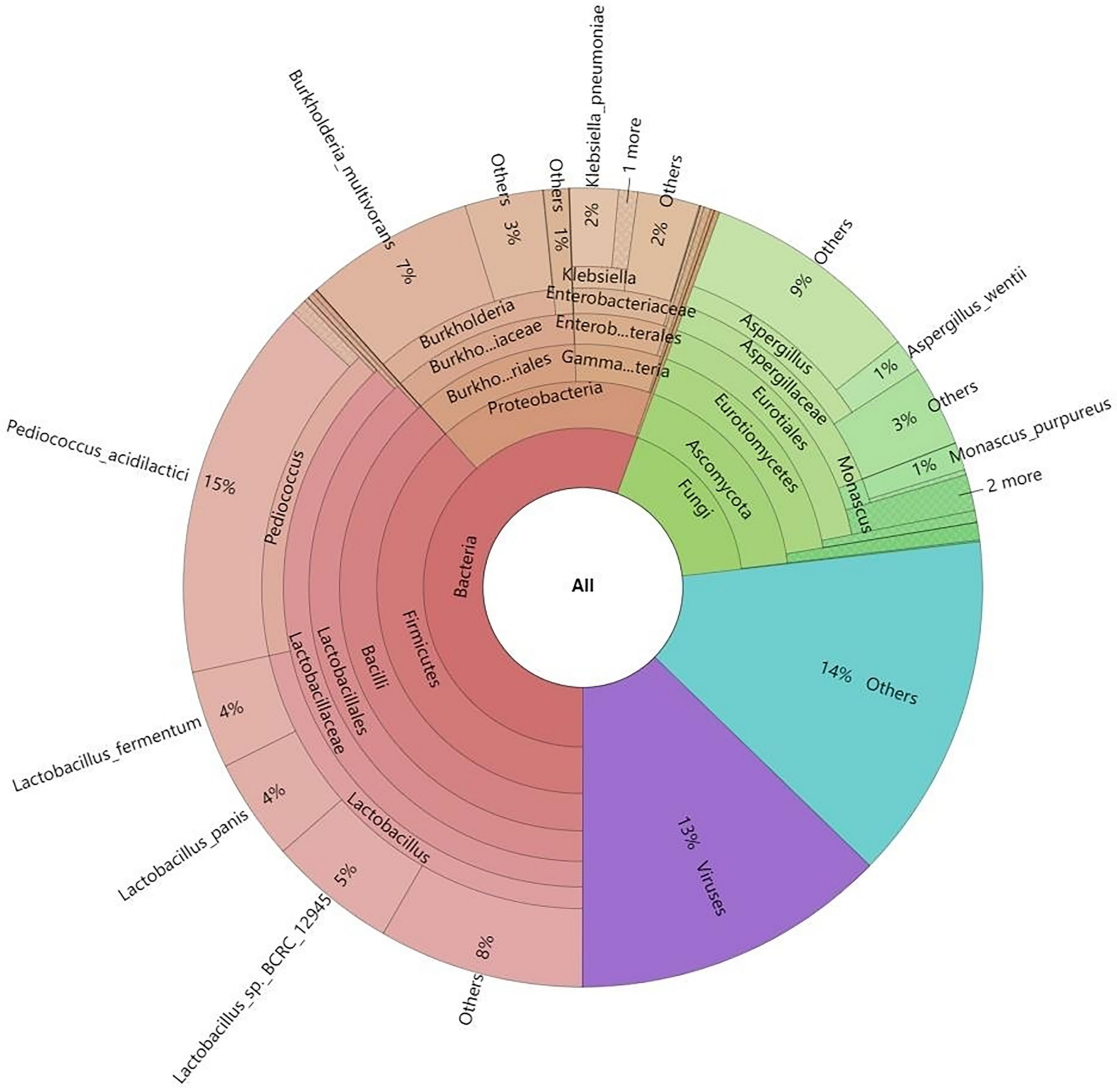
Krona diagram of the microbial community of red vinasse acid.

The red vinasse acid sample’s genome had a length of 75, 320, and 253 bp, 39,965 sequences, and a G + C content of 50.46 percent. Red vinasse acid gene species abundance annotation revealed 63 phyla, 782 genera, and 2,609 microorganisms. There are three groups of advantage bacteria among them with relative contents above 1% at the phylum level, seven groups at the genus level, and nine groups at the species level (Figure 1).

At the phylum level, *Firmicutes*, *Proteobacteria*, and *Ascomycota* were the dominant phyla, with relative contents of 38.44, 16.90, and 16.78%, respectively, accounting for 72.12% of the total content. At the genus level, the dominant genera include *Lactobacillus*, *Pediococcus*, *Aspergillus*, *Burkholderia*, *Klebsiella*, *Monascus*, and *Penicillium*, with relative contents of 21.59, 16.1, 10.25, 10.02, 2.74, 1.33, and 1.06%, respectively, accounting for 63.13% of the total content. At the species level, there are nine dominant strains with relative content of more than 1%, including *Pediococcus acidilactici*, *Burkholderia multivorans*, *Lactobacillus* sp. *BCRC 12945*, *Lactobacillus panis*, *Lactobacillus fermentum*, *Lactobacillus phage Bacchae*, *Klebsiella pneumoniae*, *Aspergillus wentii*, and *Monascus purpureus*. The relative contents were 15.47, 6.84, 5.19, 4.11, 3.97, 2.08, 1.96, 1.36, and 1.12%, respectively.

### Annotation analysis of common database

#### Annotation analysis of eggNOG database

**Figure 2:**
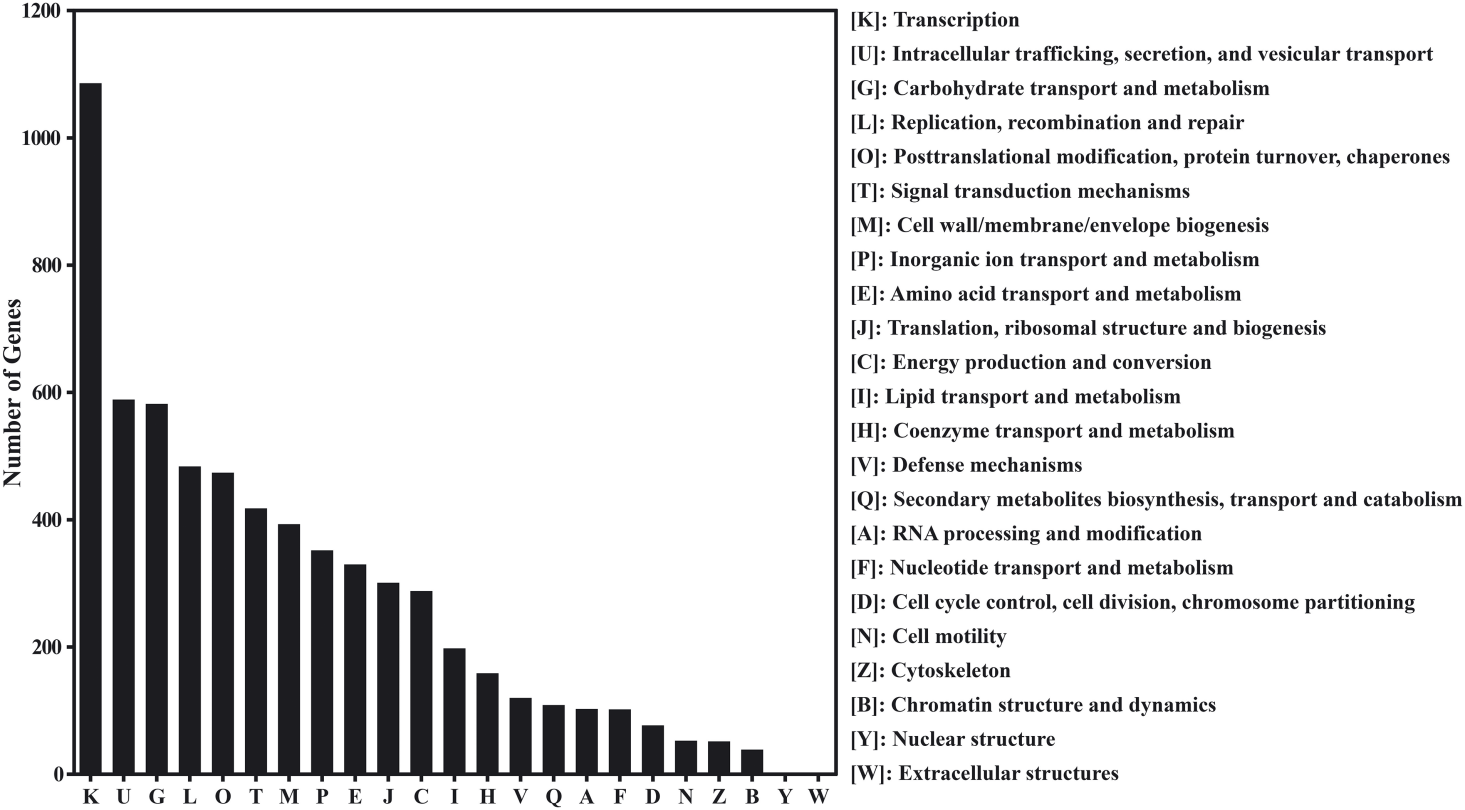
The evolutionary genealogy of genes: Non-supervised Orthologous Groups (eggNOG) gene’s functional classification in red vinasse acid.

According to the eggNOG database, 16,093 genes of red vinasse acid showed functional annotation, and these genes can be classified into 23 categories based on their functions. Remove the R (general function prediction only) and S (function unknown) parts with unknown function, in which the number of genes greater than 300 is transcription (1086), intracellular trafficking, secretion, and vesicular transport (589), carbohydrate transport and metabolism (582), replication, recombination, and repair (484), posttranslational modification, protein turnover, chaperones (474), signal transduction mechanisms (418), cell wall/membrane/envelope biogenesis (393), inorganic ion transport and metabolism (352), amino acid transport and metabolism (330), and translation, ribosomal structure, and biogenesis (301). In addition to the basic life activities of microorganisms, metabolic activities are mainly carbohydrate metabolism, inorganic ion transport and metabolism, and amino acid transport and metabolism (Figure 2).

#### Annotation analysis of KEGG database

**Figure 3:**
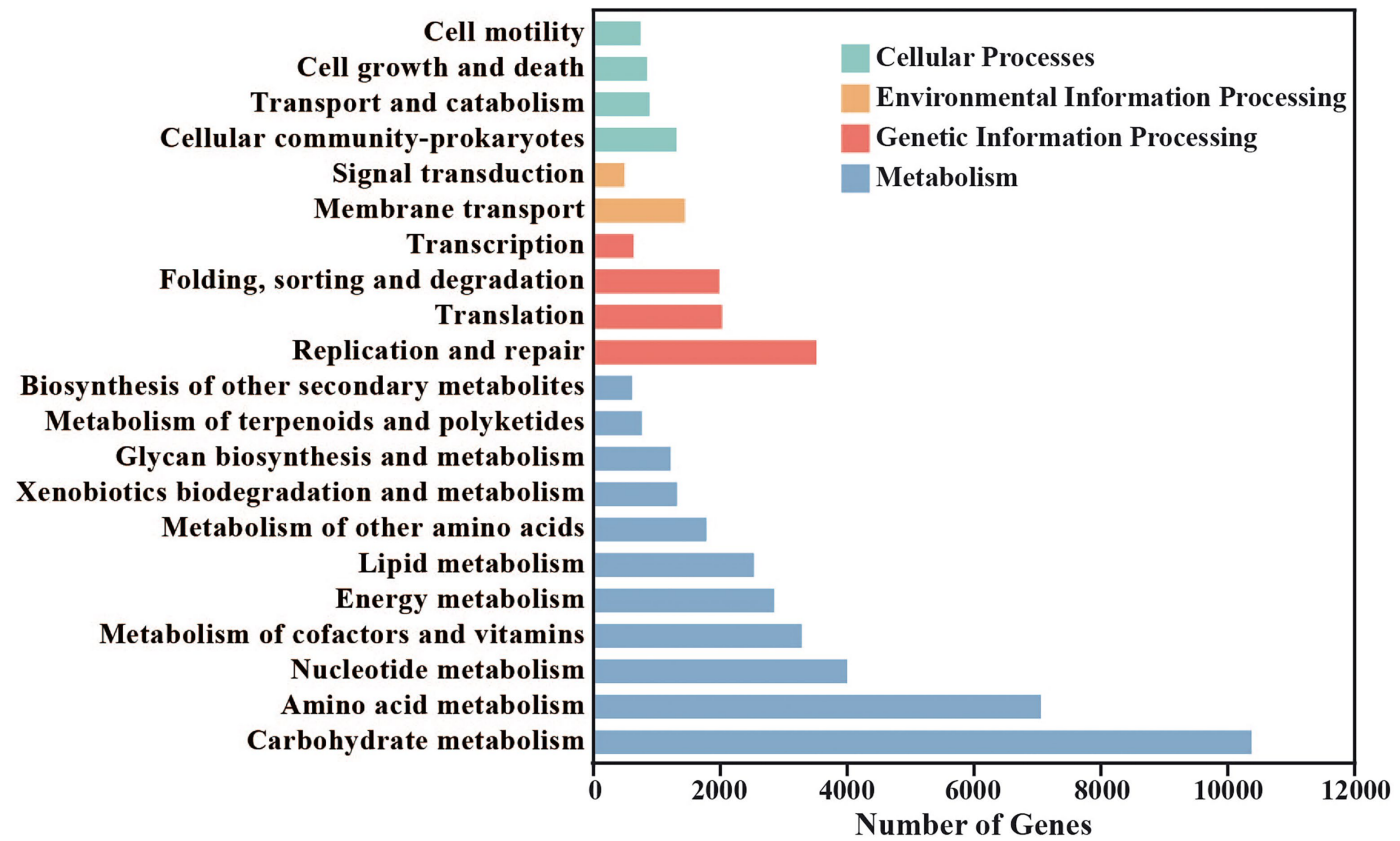
Statistics of Kyoto Encyclopedia of Genes and Genomes (KEGG) metabolic pathway in red vinasse acid.

49,652 different genes in red vinasse acid were annotated, belonging to 21 KEGG pathways in four categories of the KEGG database (Figure 3). The four categories are: metabolism (35,782, 72.07%), genetic information processing (8,272, 16.66%), cellular processes (3,670, 7.39%), and environmental information processing (1,928, 3.88%). Carbohydrate metabolism (10,375), amino acid metabolism (7,057), nucleotide metabolism (4,005), replication and repair (3,516), metabolism of cofactors and vitamins (3,284), energy metabolism (2,849), lipid metabolism (2529), and translation (2,029) are among the eight KEGG pathways that enrich differentially expressed genes (2,029). The two most significant metabolic processes among them are glucose and amino acid metabolism, which are identical to the findings of eggNOG’s functional annotation.

#### Annotation analysis of CAZy database

**Figure 4:**
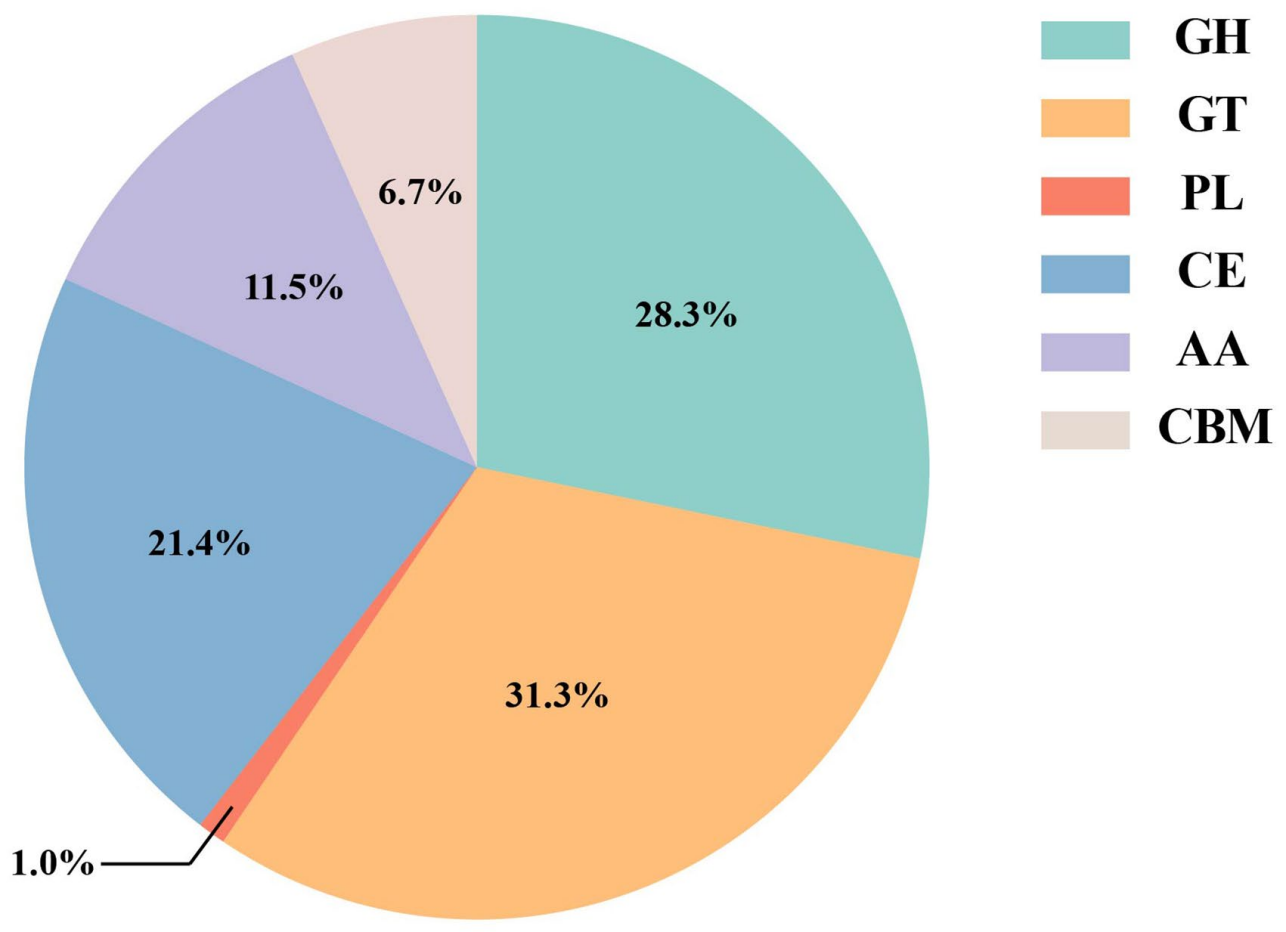
Carbohydrate-active enzymes database (CAZy) statistics in red vinasse acid.

The CAZy database can divide different types of carbohydrate-active enzymes into six protein families, including glycoside hydrolases (GHs), glycosyl transferases (GTs), polysaccharide lyases (PLs), carbohydrate esterases (CEs), carbohydrate-binding modules (CBMs), and auxiliary activities (AAs). Compared with the CAZy database, a total of 3,328 carbohydrate-active enzymes were identified (Figure 4), of which glycosyl transferases (GTs, 31.3%) and glycoside hydrolases (GHs, 28.3%) were the most. Then, carbohydrate esterases (CEs, 21.4%), auxiliary activities (AAs, 11.5%), and carbohydrate-binding modules (CBMs, 6.7%). Polysaccharide lyases (PLs, 1.0%) were the least.

### Gene analysis of glucose metabolism

#### Sugar transport system

In the sugar transport system, mannitol, fructose, mannose, and sucrose can be transported by PTS (phosphotransferase system) simultaneously. A total of 27, 49, 34, and 17 control genes were found in red vinasse acid, respectively, indicating that the ability of red vinasse acid microorganisms to transport different sugars was different during fermentation, and the ability of microorganisms to transport fructose was strong during fermentation. Lactose and maltose can be transported through the PTS transport system, ABC transport protein, and permease. One PTS transport system for lactose and maltose was found in the red vinasse acid gene, but the ABC transport protein and permease gene for lactose and maltose were not found, indicating that the transport of lactose and maltose by microorganisms in red vinasse acid was mainly carried out through the PTS transport system. Galacitol can only be transported through the PTS transport system, and 16 related genes were found in red vinasse acid. Gluconate, fructose, and oligosaccharides can also be used to enter the strain cells by using relevant permeability proteins, and 10, 0, and 14 genes were retrieved respectively, indicating that fructose is mainly transported by microorganisms in red vinasse acid through the PTS transport system (Table 1).

**Table 1:** Gene analysis of the sugar transport system in red vinasse acid.

Sugar	Transporter	Gene number
Mannitol	PTS-Gtl-EIIA/B	27
Fructose	PTS-Gfr-EIIA/B	49
Mannose	PTS-Gan-EIIA/B	34
Sucrose	PTS-Scr-EIIA/B	17
Lactose	PTS-Lac-EIIB	1
Maltose	PTS-Mal-EIIB	1
Galactitol	PTS-Gat-EIIA/B	16
Glucose	PTS-Glc/v-EIIA/B	22
Galactose	PTS-Gal-EIIA/B	3
Gluconate	permease protein	10
Oligosaccharides	permease protein	14

#### Transformation of D-mannitol 1-phosphate

The PTS transport system phosphorylates sugars before they are delivered to cells. Mannitol was phosphorylated while being transported by the PTS system, and after reaching the cells, D-mannitol 1-phosphate was eventually produced. The latter is often transformed by mannitol-1-phosphate 5-dehydrogenase (EC: 1.1.1.17) into D-Fructose 6-phosphate, an intermediate glycolysis product. In conjunction with Tables 1, 2, red vinasse acid has 14 mannitol-1-phosphate 5-dehydrogenase genes and the mannitol PTS transport system, allowing mannitol to be transformed into the intermediate of the glycolysis pathway known as D-Fructose 6-phosphate. Table 2 demonstrates that there are seven species of bacteria and one species of fungi at the genus level, with *Lactobacillus* being the predominate genus and playing a significant role in the transfer of mannitol (Table 2; Chen et al., 2014).

**Table 2:** Genes encoding enzymes involved in conversion sugar into intermediates of glycolysis pathway.

	Enzyme	K0 number	EC number	Gene name	Gene number	Annotated microorganisms
Transformation of D-mannitol 1-phosphate	Mannitol-1-phosphate 5-dehydrogenase	K00009	EC: 1.1.1.17	mtlD	14	*Lactobacillus* (*5*), *Franconibacter* (*2*), *Paenibacillus* (*2*), *Unclassified*, *Pantoea*, *Klebsiella*, *Cronobacter*, and *Rasamsonia*
Transformation of D-Fructose 1-phosphate	6-phosphofructokinase 1	K00850	EC: 2.7.1.11	PFK1	24	*Millerozym* (*6*), *Unclassified* (*3*), *Lactobacillus* (*3*), *Paenibacillus* (*2*), *Klebsiella* (*2*), *Pantoea* (*2*), *Aspergillus* (*2*), *Kluyveromyces*, *Pediococcus*, *Penicillium*, and *Chryseobacterium*
	Fructose-bisphosphate aldolase, class I	K11645	EC: 4.1.2.13	ALDO	7	*Franconibacter* (*2*), *Cronobacter* (*2*), *Pantoea*, *Klebsiella*, and *Acetobacter*
	Fructose-bisphosphate aldolase, class II	K01624	EC: 4.1.2.13	FBA	29	*Lactobacillus* (*7*), *Unclassified* (*5*), *Klebsiella* (*3*), *Paenibacillus* (*2*), *Pantoea* (*2*), *Franconibacter* (*2*), *Pediococcus*, *Cronobacter*, *Burkholderia*, *Chryseobacterium*, *Acinetobacter*, *Millerozyma*, *Metarhizium*, *Aspergillus*, and *Byssochlamys*
	Triosephosphate isomerase (TIM)	K01803	EC: 5.3.1.1	TPI	33	*Lactobacillus* (*15*), *Burkholderia* (*2*), *Klebsiella* (*2*), *Millerozyma* (*2*), *Aspergillus* (*2*), *Luteibacter*, *Pantoea*, *Pediococcus*, *Acetobacter*, *Unclassified*, *Franconibacter*, *Acinetobacter*, *Cronobacter*, *Cladophialophora*, and *Zygosaccharomyces*
	Triose kinase	K00863	EC: 2.7.1.28	DAK	13	*Millerozyma* (*7*), *Burkholderia* (*2*), *Aspergillus* (*2*), *Cronobacter*, and *Byssochlamys*
Transformation of D-mannose-6-phosphate	Mannose-6-phosphate isomerase	K01809	EC: 5.3.1.8	manA	26	*Lactobacillus* (*4*), *Franconibacter* (*4*), *Klebsiella* (*4*), *Cronobacter* (*3*), *Pediococcus* (*2*), *Lactobacillus* (*2*), *Aspergillus* (*2*), *Pantoea*, *Unclassified*, *Paenibacillus*, *Penicillium*, and *Millerozyma*
Transformation of sucrose-6-phosphate	beta-fructofuranosidase	K01193	EC: 3.2.1.26	sacA	20	*Lactobacillus* (*9*), *Klebsiella* (*6*), *Franconibacter* (*4*), and *Cronobacter*
Transformation of lactose 6-phosphate	6-phospho-beta-galactosidase	K01221	EC: 3.2.1.85	lacG	0	*——*
Transformation of gluconate	Gluconokinase	K00851	EC: 2.7.1.12	gntK	28	*Lactobacillus* (*14*), *Klebsiella* (*2*), *Pediococcus* (*2*), *Paenibacillus* (*2*), *Burkholderia* (*2*), *Pantoea*, *Franconibacter*, *Cronobacter*, *Millerozyma* (*2*), and *Aspergillus*
	6-phosphogluconate dehydrogenase	K00033	EC: 1.1.1.44 1.1.1.343	PGD	70	*Lactobacillus* (*14*), *Unclassified* (*23*), *Klebsiella* (*8*), *Paenibacillus* (*4*), *Pediococcus* (*3*), *Millerozyma* (*3*), *Burkholderia* (*2*), *Cronobacter* (*2*), *Pantoea* (*2*), *Chryseobacterium* (*2*), *Acetobacter*, *Luteibacter*, *Franconibacter*, *Kluyveromyces*, *Talaromyces*, *Aspergillus*, and *Rasamsonia*
	Ribulose-phosphate 3-epimerase	K01783	EC: 5.1.3.1	RPE	26	*Lactobacillus* (*8*), *Stenotrophomonas* (*2*), *Burkholderia* (*2*), *Millerozyma* (*2*), *Franconibacter*, *Pantoea*, *Acinetobacter*, *Acetobacter*, *Klebsiella*, *Ideonella*, *Luteibacter*, *Pediococcus*, *Sphingomonas*, *Cronobacter*, *Aspergillus*, and *Kluyveromyces*
	transketolase	K00615	EC: 2.2.1.1	tktA	85	*Lactobacillus* (*21*), *Unclassified* (*14*), *Klebsiella* (*12*), *Franconibacter* (*10*), *Burkholderia* (*6*), *Pantoea* (*3*), *Luteibacter* (*3*), *Acetobacter* (*2*), *Paenibacillus* (*2*), *Acinetobacter* (*2*), *Enterobacter* (*2*), *Cronobacter* (*2*), *Millerozyma* (*2*), *Kozakia*, *Stenotrophomonas*, *Sphingomonas*, and *Aspergillus*
Transformation of maltose	Maltose phosphorylase	K00691	EC: 2.4.1.8	mapA	18	*Lactobacillus* (*16*), *Paenibacillus* (*2*)
Transformation of lactose	Beta-galactosidase	K01190 K12308	EC: 3.2.1.23	lacZ	65	*Lactobacillus* (*29*), *Burkholderia* (*6*), *Paenibacillus* (*6*), *Franconibacter* (*5*), *Klebsiella* (*4*), *Kluyveromyces* (*3*), *Pantoea* (*2*), *Chryseobacterium* (*2*), *Pediococcus* (*2*), *Aspergillus* (*2*), *Sphingomonas*, *Cronobacter*, *Luteibacter*, and *Unclassified*
Transformation of lactose and galactose	Galactokinase	K00849	EC: 2.7.1.6	galK	20	*Lactobacillus* (*7*), *Pediococcus* (*2*), *Millerozyma* (*2*), *Saccharomyces*, *Aspergillus* (*2*), *Pantoea*, *Enterobacter*, *Franconibacter*, *Chryseobacterium*, *Cronobacter*, and *Klebsiella*
	UDP glucose-hexose-1-phosphate uridylyltransferase	K00965	EC: 2.7.7.12	galT	17	*Lactobacillus* (*6*), *Franconibacter* (*3*), *Unclassified* (*2*), *Pantoea*, *Paenibacillus*, *Cronobacter*, *Pediococcus*, *Millerozyma*, and *Aspergillus*

#### Transformation of D-fructose 1-phosphate

Fructose was finally transported into cells through the PTS system to form D-Fructose 1-phosphate, which was generally converted into the intermediate product of glycolysis D-glyceraldehyde 3-phosphate through three pathways. There are three combinations of key enzymes, namely, phosphofructokinase (EC: 2.7.1.11) and fructose-2-phosphate aldolase, fructose-bisphosphate aldolase (EC: 4.1.2.13) and triosephosphate isomerase (EC: 5.3.1.1), and fructose-2-phosphate aldolase and triose kinase (EC: 2.7.1.28). Combined with Tables 1, 2, red vinasse acid contains fructose PTS transport system, 36 fructose-bisphosphate aldolase and 33 triphosphate isomerase genes, which can convert intracellular D-Fructose 1-phosphate into D-glyceraldehyde 3-phosphate by the second pathway. Fructose-bisphosphate aldolase (EC: 4.1.2.13) comes in two varieties: fructose-bisphosphate aldolase class I and class II, as indicated in Table 2. Fructose-bisphosphate aldolase and class I annotated five kinds of microorganisms, and the dominant genera were *Franconibacter* and *Cronobacter*. Fructose-bisphosphate aldolase and class II annotated 15 species of microorganisms, and the dominant genus was *Lactobacillus*. Triosephosphate isomerase (EC:5.3.1.1) annotated 15 species of microorganisms, including 11 species of bacteria and four species of fungi. The dominant genera of bacteria were *Lactobacillus* and the dominant genera of fungi were *Millerozyma* and *Aspergillus*. Therefore, *Lactobacillus* plays a major role in the transport of fructose.

#### Transformation of D-mannose-6-phosphate

Mannatose was transported to cells through the PTS system to form D-mannose-6-phosphate, which was directly converted into the intermediate product β-D-Fructose 6 phosphate in the glycolysis pathway catalyzed by mannose-6-phosphate isomerase (EC: 5.3.1.8). Combining Tables 1, 2, it is known that red vinasse acid contains a mannose PTS transport system and 26 mannose-6-phosphate isomerase genes, so mannose can be converted into β-D-Fructose 6 phosphate, which is the intermediate of the glycolysis pathway. As shown in Table 2, nine species of bacteria and three species of fungi were annotated in the transport process. The dominant bacterial genera were *Franconibacter*, *Klebsiella*, and *Lactobacillus*, and the dominant genus of fungi was *Aspergillus*, indicating that the transport of mannose was mainly completed by the above microorganisms.

#### Transformation of sucrose-6-phosphate

Sucrose was transported to cells by the PTS system to form sucrose 6-phosphate, which was then converted to D-glucose 6-phosphate and β-D-fructose under the catalysis of β-fructofuranosidase (EC: 3.2.1.26). Combined with Tables 1, 2, red vinasse acid contains the PTS transport system of sucrose and encodes 20 genes of β-fructofuranosidase, so red vinasse acid can be transported to intracellular by extracellular sucrose to further produce D-glucose 6-phosphate and β-D-fructose. As shown in Table 2, four kinds of microorganisms are noted. All of them are bacteria, and the dominant genus is *Lactobacillus*.

#### Transformation of lactose 6-phosphate

The product of lactose transported to cells *via* the PTS system is 6-phosphate lactose, which is catalyzed by 6-phospho-β-galactosidase (EC: 3.2.1.85) to form β-D-glucose and D-galactose-6-phosphate. Combined with Tables 1, 2, the PTS transport system contained lactose, but the gene encoding 6-phospho-β-galactosidase was not found, so the transport of lactose could not be realized by red vinasse acid.

#### Transformation of gluconate

Gluconate transported to the cells can further form the intermediate substance β-D-fructose 6-phosphate in the glycolysis pathway, but this process needs to be catalyzed by four enzymes: glucokinase (EC: 2.7.1.12), 6-phosphogluconate dehydrogenase (EC:1.1.1.44 1.1.1.343), ribulose-phosphate 3-epimerase (EC: 5.1.3.1), and transketolase (EC: 2.2.1.1). The control genes of four enzymes were found in red vinasse acid, and the number of genes was 28, 70, 26, and 85, respectively, so red vinasse acid can use extracellular gluconate to further produce β-D-fructose-6-phosphate and participate in the pathway of glycolysis. As shown in Table 2, glucokinase annotated 10 species of microorganisms, including eight species of bacteria and two species of fungi, and the dominant genus was *Lactobacillus*. Twelve species of bacteria, with Unclassified and *Lactobacillus* as the most prevalent genera, and five species of fungi, with *Millerozyma* as the most prevalent genus, were found to contain 6-phosphate gluconate dehydrogenase. Ribulose-3-phosphate isomerase annotated three species of fungi and 13 species of bacteria. *Millerozyma* was the main fungus genus, whereas Lactobacillus was the leading genus of bacteria. The predominant genus among the 17 microorganism species for which transketolase was annotated was *Lactobacillus*. As a result, *Lactobacillus* is crucial to the transfer of gluconate.

#### Transformation of maltose

After entering the bacteria cells, maltose can be converted into D-glucose under the action of maltose phosphorylase (EC: 2.4.1.8) and enter the glycolysis pathway directly. 18 genes of maltose phosphorylase are encoded in red vinasse acid, so maltose can be converted into D-glucose, an intermediate product of the glycolysis pathway. As shown in Table 2, maltose phosphorylase was annotated to two microorganisms, and *Lactobacillus* was the dominant genus.

#### Transformation of lactose

Lactose enters the cell through the PTS transport system and produces glycolysis intermediates through two pathways (Table 2). The lactase transferred into the cell was hydrolyzed into α-D-glucose and D-glucose-1-phosphate by pathways I and II. In pathway I, there are 70 genes encoding β-galactosidase (EC: 3.2.1.23), and in pathway II, the number of genes encoding β-galactosidase (EC: 3.2.1.23)/galactokinase (EC: 2.7.1.6)/UDP- glucose-hexose-1-phosphate uridylyltransferase (EC: 2.7.7.12) is 70, 20, and 17, respectively. Therefore, the intracellular lactose in red vinasse acid can be hydrolyzed into α-D-glucose and D-glucose-1-phosphate by pathways I and II. As shown in Table 2, β-galactosidase, galactokinase, and UDP- glucose-hexose-1-phosphate uridine invertase were annotated to 14, 11, and 9 species of microorganisms, respectively, and all the dominant bacteria were *Lactobacillus*.

#### Transformation of galactose

Galactose could transform D-glucose 1-phosphate under the catalysis of galactokinase (EC: 2. 7. 1. 6) and UDP-glucose-hexose-1-phosphate uridylyltransferase (EC: 2.7.7.12), and 20 and 17 coding genes were found and annotated for 11 and nine species of microorganisms, respectively. All the dominant bacteria were *Lactobacillus*. And red vinasse acid contains the PTS transport system of galactose, so galactose can be converted into D-glucose 1-phosphate.

### Amino acid flavor formation pathway

The pathway of amino acid flavor formation means that amino acids are transformed into corresponding ketoacids by a transaminase. Which are unstable, converted into corresponding aldehydes, further dehydrogenated to alcohols, and then esterified to corresponding esters. These metabolites are important flavor components in food.

#### Transaminase

**Figure 5:**
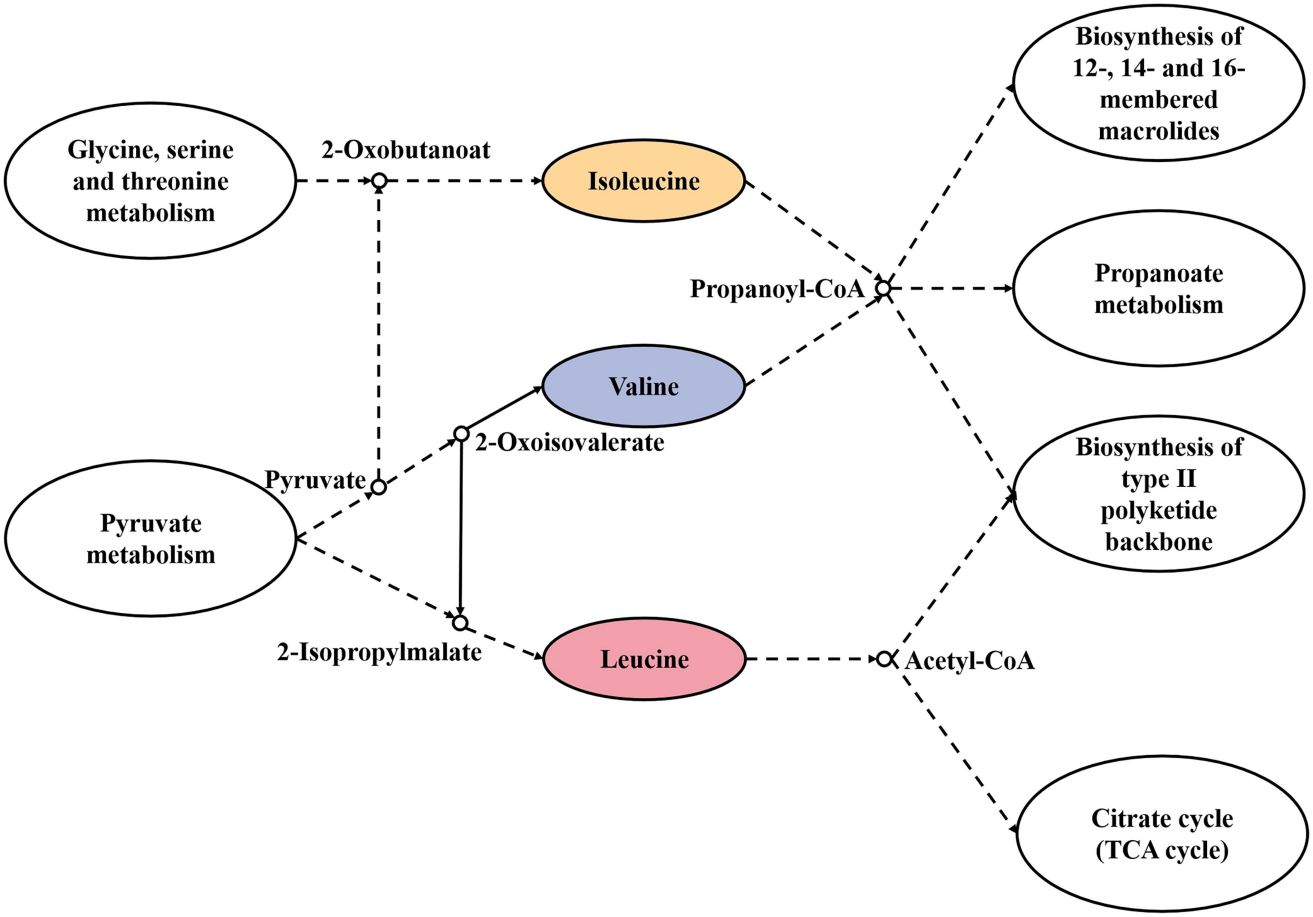
Synthesis and metabolic pathway of branched-chain amino acids.

**Figure 6:**
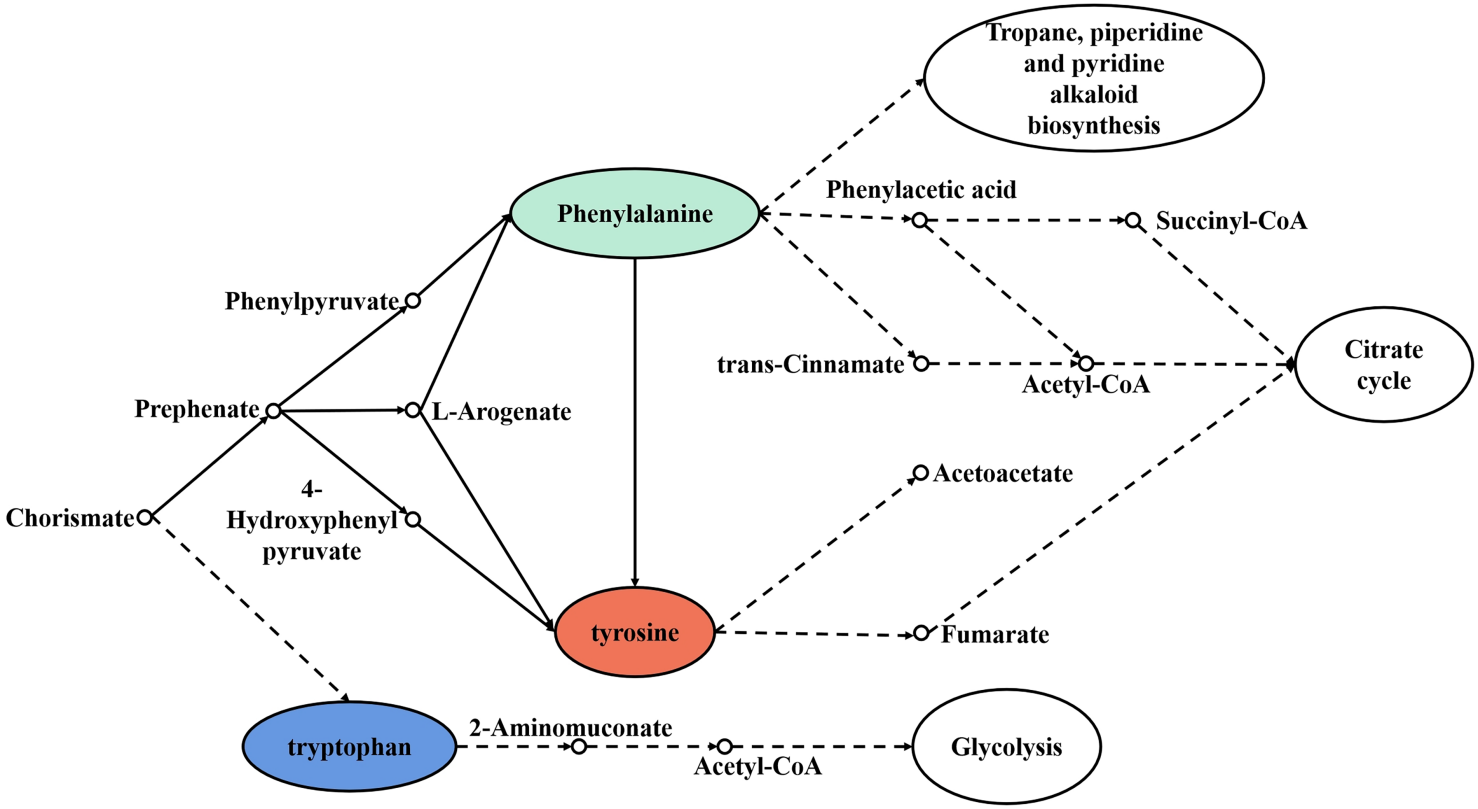
Synthesis and metabolic pathway of aromatic amino acids.

The transamination of branched-chain amino acids, aromatic amino acids, and methionine can be catalyzed by different transaminases. Branched-chain amino acid aminotransferase (BCAT) has activity for branched-chain amino acids and methionine, while aromatic-amino-acid transaminase (ArAT) has catalytic activity for aromatic amino acids, leucine, and methionine. As shown in Table 3, 32 BCAT genes (ilvE) were identified, and 15 kinds of microorganisms were annotated with red vinasse acid genes, and the dominant strain was *Aspergillus*. And 27 ArAT genes (tyrB, ARO8, and ARO93) were found, which were, respectively, annotated to seven, six, and two kinds of microorganisms. The dominant bacterial genera were *Burkholderia*, *Debaryomyces*, and *Millerozyma*, respectively. According to the findings, the key players in the fermentation of red vinasse acid were *Aspergillus*, *Burkholderia*, *Debaryomyces*, and *Millerozyma*, which catalyzed the synthesis of branched-chain amino acids, aromatic amino acids, and methionine. Figures 5, 6 respectively depict the metabolic and synthesis processes for aromatic amino acids and branched-chain amino acids.

**Table 3:** Genes of encoding enzymes involved in transamination pathway in red vinasse acid.

Abbreviation	Gene name	KO number	EC number	Gene number	Functional description	Annotated microorganisms
BcAT	*ilvE*	K00826	EC: 2.6.1.42	32	Branched-chain amino acid aminotransferase	*Aspergillus* (*5*), *Burkholderia* (*4*), *Lactobacillus* (*3*), *Cronobacter* (*3*), *Klebsiella* (*3*), *Acetobacter* (*2*), *Luteibacter* (*2*), *Chryseobacterium* (*2*), *Millerozyma* (*2*), *Phialosimplex*, *Stenotrophomonas*, *Staphylococcus*, *Pantoea*, *Franconibacter*, and *Acinetobacter*
ArAT	*tyrB*	K00832	EC: 2.6.1.57	17	Aromatic-amino-acid transaminase	*Burkholderia* (*5*), *Acinetobacter* (*3*), *Klebsiella* (*3*), *Unclassified* (*2*), *Luteibacter* (*2*), *Stenotrophomonas*, and *Cronobacter*
ArAT	*ARO8*	K00838	EC: 2.6.1.57 2.6.1.27 2.6.1.5	7	Aromatic amino acid aminotransferase I	*Debaryomyces* (*2*), *Clavispora*, *Wickerhamiella*, *Unclassified*, *Penicilliopsis*, and *Aspergillus*
ArAT	*ARO9*	K05821	EC: 2.6.1.58 2.6.1.28	3	Aromatic amino acid aminotransferase II	*Millerozyma* (*2*), *Kluyveromyces*
PTA	*pta*	K00625	EC: 2.3.1.8	14	Phosphate acetyltransferase	*Lactobacillus* (*8*), *Burkholderia* (*3*), *Pediococcus*, *Acetobacter*, and *Enhydrobacter*
PTA	*pta*	K13788	EC: 2.3.1.8	13	Phosphate acetyltransferase	*Unclassified* (*6*), *Acinetobacter* (*3*), *Pantoea*, *Klebsiella*, *Franconibacter*, and *Cronobacter*
ACK	*ackA*	K00925	EC: 2.7.2.1	33	Acetate kinase	*Lactobacillus* (*16*), *Burkholderia* (*6*), *Pediococcus* (*3*), *Unclassified* (*2*), *Paenibacillus*, *Acetobacter*, *Franconibacter*, *Acinetobacter*, *Aspergillus*, and *Penicillium*
AlcDH	*adh*	K00001	EC: 1.1.1.1	13	Alcohol dehydrogenase	*Lactobacillus* (*7*), *Burkholderia* (*2*), *Acinetobacter*, *Klebsiella*, *Enterobacter*, and *Pediococcus*
AlcDH	*adh2*	K18369	EC: 1.1.1.-	3	Alcohol dehydrogenase	*Burkholderia* (*2*), *Aspergillus*
AlcDH	*yiaY*	K13954	EC: 1.1.1.1	7	Alcohol dehydrogenase	*Cronobacter* (*3*), *Franconibacter* (*2*), *Lactobacillus*, and *Acinetobacter*
AlcDH	*AKR1A1*	K00002	EC: 1.1.1.2	3	Alcohol dehydrogenase (NADP+)	*Millerozyma* (*3*)
AlcDH	*exaA*	K00114	EC: 1.1.2.8	3	Alcohol dehydrogenase (cytochrome c)	*Burkholderia* (*2*), and *Acetobacter*
AlcDH	*adhP*	K13953	EC: 1.1.1.1	33	Alcohol dehydrogenase, propanol-preferring	*Lactobacillus* (*14*), *Millerozyma* (*4*), *Burkholderia* (*3*), *Pediococcus*, *Asticcacaulis*, *Rhizobium*, *Unclassified*, *Franconibacter*, *Klebsiella*, *Pantoea*, *Fonsecaea*, *Aspergillus*, *Monascus*, *Penicillium*, and *Byssochlamys*,
AlcDH	*yqhD*	K08325	EC: 1.1.-. -	8	NADP-dependent alcohol dehydrogenase	*Klebsiella* (*3*), *Cronobacter* (*2*), *Franconibacter* (*2*), and *Chryseobacterium*
AlcDH	*adhC*	K00121	EC: 1.1.1.284 1.1.1.1	13	S-(hydroxymethyl)glutathione dehydrogenase / alcohol dehydrogenase	*Acetobacter* (*2*), *Franconibacter* (*2*), *Unclassified* (*2*), *Paenibacillus*, *Burkholderia*, *Pantoea*, *Luteibacter*, *Stenotrophomonas*, *Byssochlamys*, and *Millerozyma*,
AldDH	*adhE*	K04072	EC: 1.2.1.10 1.1.1.1	23	Acetaldehyde dehydrogenase / alcohol dehydrogenase	*Lactobacillus* (*10*), *Franconibacter* (*5*), *Unclassified* (*3*), *Pantoea* (*2*), *Paenibacillus* (*2*), and *Cronobacter*
AldDH	*feaB*	K00146	EC: 1.2.1.39	9	Phenylacetaldehyde dehydrogenase	*Burkholderia* (*3*), *Klebsiella* (*3*), *Unclassified* (*2*), and *Acinetobacter*
AldDH	*mhpF*	K04073	EC: 1.2.1.10	6	Acetaldehyde dehydrogenase	*Burkholderia* (*3*), *Unclassified*, *Escherichia*, and *Klebsiella*
AldDH	*bphJ*	K18366	EC: 1.2.1.10 1.2.1.87	2	Acetaldehyde/propanal dehydrogenase	*Burkholderia* (*2*)
EstA	*aes*	K01066	EC: 3.1.1.-	27	Acetyl esterase	*Burkholderia* (*6*), *Klebsiella* (*4*), *Unclassified* (*4*), *Lactobacillus* (*3*), *Citrobacter* (*3*), *Franconibacter* (*2*), *Chryseobacterium*, *Paenibacillus*, *Acetobacter*, *Enterobacter*, and *Acinetobacter*

#### Ketoacid invertase

Keto acids can be converted in three ways. The first way is that keto acids can be directly converted into carboxylic acids by oxidative decarboxylation. In this pathway, keto acid dehydrogenase (KaDH), phosphate acetyltransferase (PTA), and acetate kinase (ACK) showed activity on the matrix of branched-chain amino acids. As shown in Table 3, there are genes encoding PTA (27) and ACK (33) in the genomic DNA of red vinasse acid, which were annotated to 11 and 7 microorganisms, respectively, indicating that keto acids of the above amino acids can be converted into corresponding carboxylic acids in the process of red vinasse acid fermentation, and the dominant genus was *Lactobacillus*.

The second pathway is that keto acids are decomposed into carboxylic acids by carboxylic acid dehydrogenase (HycDH). HycDH has two stereospecific enzymes, D-carboxylate dehydrogenase (D-HycDH), and L-carboxylate dehydrogenase (L-HycDH). At present, although it cannot be proved that this approach can directly lead to the formation of flavored substances, it can reduce the synthesis of flavored substances by shunting the precursors of flavor substances into non-flavored products. These two carboxylic acid dehydrogenases were not found in the genomic DNA of red vinasse acid, so the overall flavor will not be reduced because of the above pathways.

The third way is the conversion of α-ketoacid to corresponding aldehydes by α-ketoacid decarboxylase (KdcA). The gene encoding keto acid decarboxylase was not found in red vinasse acid, which indicated that the microorganisms in red vinasse acid could not carry out the latter two forms of transformation, and the first keto-acid transformation was mainly used in the fermentation process, and *Lactobacillus* was the main function.

#### Alcohol and aldehyde dehydrogenase

Aldehyde dehydrogenase and alcohol dehydrogenase can catalyze the conversion of aldehydes (alcohols) into corresponding alcohols and carboxylic acids, and the control genes are aldehyde dehydrogenase (AldDH) and alcohol dehydrogenase (AlcDH), respectively. In the red vinasse acid gene, 83 genes encoding alcohol dehydrogenase (AlcDH) and 40 genes encoding aldehyde dehydrogenase (AldDH) were found. Alcohol dehydrogenase (AlcDH) includes adh, adh2, yiaY, AKR1A1, exaA, adhP, yqhD, and adhC. It is noted that the dominant genus is *Lactobacillus*, and the fungus *Millerozyma* also plays an important role. Aldehyde dehydrogenase (AldDH) includes adhE, feaB, mhpF, and bphJ, and the dominant genus is *Lactobacillus*. Therefore, in the process of catalyzing aldehydes (alcohols) into corresponding alcohols and carboxylic acids, *Lactobacillus* was the main microorganism.

## Discussion

Red vinasse acid has a rich aroma and unique flavor, which is closely related to its complex microbial metabolic activities. It has been reported that in fermented food, the different dynamic patterns of free sugars and amino acids imply different co-metabolisms, for example, free sugars might derive from polysaccharides through hydrolysis and be consumed by lactic acid bacteria, whereas amino acids produced through proteolysis of proteins are consumed by fungi (Mouritsen et al., 2017).

In this study, macrogenomic analysis revealed the microbial diversity and functional properties of the microbial community. We annotated the species abundance of the red vinasse acid gene, and the dominant genera included *Lactobacillus*, *Pediococcus*, *Aspergillus*, *Burkholderia*, *Klebsiella*, *Monascus*, and *Penicillium* (Figure 1). *Lactobacillus* can produce lactic acid and a variety of antibacterial substances. *Lactobacillus* intracellular carbohydrate active enzymes are specific (Swanson et al., 2020). The amount of lactic acid produced during fermentation has an impact on the quality of the fermentation. In addition to improving the nutritional value and quality of food, *Pediococcus* also has great resistance to acid and high temperatures. Additionally, *Pediococcus* displays biological properties that fight cancer and lower cholesterol (Zhang et al., 2019). *Monascus* is the main beneficial fungus of red vinasse acid fermentation, which gives red vinasse acid pigment and a unique flavor. Due to the lack of sterilization technology in the production of traditional red vinasse acid, the community information of microorganisms in red vinasse acid is complex and diverse, so in addition to beneficial microbes such as *Lactobacillus and Monascus*, there are still a small number of spoilage microbes and detrimental microbes in the red vinasse acid, such as *Burkholderia* and *Klebsiella.* The comparative analyses that can be performed are limited by the relatively few reports on the microbial diversity of red vinasse acid at the species level, and our macrogenomic analysis of red vinasse acid has enriched the information on the specific category information of microbial diversity of red vinasse acid. Compared with the eggNOG and KEGG databases, 16,093 and 49,652 genes were annotated, respectively. The main metabolic activities were amino acid metabolism and carbohydrate metabolism (Figures 2, 3). Glycosyl transferases and glycoside hydrolases were the most abundant in the CAZy database analysis (Figure 4). Glycoside hydrolases hydrolyze the glycosidic bonds of various sugar-containing compounds such as oligosaccharides and polysaccharides by internal or external digestion and generate monosaccharides, oligosaccharides, or sugar complexes, thus playing an important role in the synthesis of oligosaccharides and aromatic glycosides, and the glycosylation of amino acids and peptides. Glycosyl transferases catalyze the binding of activated sugars to non-receptor molecules, such as proteins, oligosaccharides, lipids, and small molecules (Chen et al., 2013; Cai et al., 2018; Huang et al., 2018). The abundant glycoside hydrolase and glycosyl transferase in red vinasse acid provide the basis for the formation, transfer, and further metabolism of monosaccharides and oligosaccharides.

In the pathway of glucose metabolism, red vinasse acid encodes the sugar transport system genes of mannitol, fructose, mannose, and sucrose, and retrieves the genes of gluconate and oligosaccharide related permeability proteins (Table 1). It also has the key enzyme genes that catalyze intracellular D-mannitol-1-phosphate, D-fructose-1-phosphate, D-mannose-6-phosphate, sucrose-6-phosphate, gluconate, maltose, lactose, and galactose, and has the basis for transforming them into glycolysis intermediates, and the main dominant bacterial genus of the glucose metabolism pathway is *Lactobacillus* (Table 2). *Lactobacillus* uses the relevant transport mechanism to transport various sugar substances from outside the cell to the cell, and then transformed into intermediate products of the glycolytic pathway through the catalysis of various related enzymes. These substances enter the glycolytic pathway and finally produce pyruvate, the precursor substance of lactic acid, through the catalysis of related enzymes. Pyruvate can be catalyzed by L-lactate dehydrogenase or D-lactate dehydrogenase to ultimately produce L-lactate or D- lactate (Kleerebezemab et al., 2000; Wang et al., 2010), resulting in a unique flavor.

In the process of amino acid flavor formation, the control genes of 32 branched-chain amino acid aminotransferase (BCAT), 27 aromatic-amino-acid transaminases (ArAT), 60 ketoacid invertase, 123 alcohol/aldehyde dehydrogenases, and 27 acetyl esterases were encoded by red vinasse acid (Table 3). Amino acids can be transformed into corresponding keto acids by transaminase, and then into corresponding aldehydes, further dehydrogenation to alcohols, and then esterification to corresponding esters, which have the basis for the formation of strong ester flavors, and the main dominant bacteria genus during amino acid flavor formation is *Lactobacillus*. Figure 5 shows the synthesis and metabolic pathway of branched-chain amino acids (Leucine, Valine, and Isoleucine), which is a crosstalk and a simplified process of the KEGG metabolic pathway ko00290 and ko00280. The solid line in the diagram represents the direct process and the dashed line represents the indirect process. The degradation of Valine and Isoleucine to Propanoyl-CoA is catalyzed by a series of enzymes, and Propanoyl-CoA then indirectly enters the following three metabolic pathways: Biosynthesis of 12-, 14-, and 16-membered macrolides, Propanoate metabolism, and Biosynthesis of type II polyketide backbone. Leucine is metabolized to Acetyl-CoA by a series of enzymes, and Acetyl-CoA enters indirectly into the Biosynthesis of type II polyketide backbone and the Citrate cycle (TCA cycle), which further functions in two metabolic pathways. Figure 6 shows the synthesis and metabolic pathway of aromatic amino acids (Phenylalanine, Tyrosine, and Tryptophan), which is a crosstalk and a simplified process of the KEGG metabolic pathway ko00400, ko00360, ko00350, and ko00380. Tryptophan is metabolically involved in the glycolytic process, Tyrosine in the tricarboxylic acid cycle, and Phenylalanine in the Tropane, piperidine, and pyridine alkaloid biosynthesis and Citrate cycle. We show the synthesis and metabolic pathways of branched-chain amino acids and aromatic amino acids in this study in the form of pictures, which are simplified to mainly describe the relationship between metabolic pathways and amino acids compared to the complete metabolic pathways in KEGG, in order to highlight the wide range and importance of amino acids in the process of biological activities, as well as to provide a targeted direction for flavor studies in amino acid metabolism.

The primary metabolic functions of red vinasse acid and the essential enzyme genes for sugar and amino acid metabolism were further discovered based on an examination of the structure and makeup of red vinasse acid bacteria. Analysis of dominant strain roles in the major metabolic pathways and further confirmation of the expression of important enzyme genes. It is anticipated that it will offer a theoretical foundation for the enhancement of red vinasse acid quality and the mining of microbial functional gene pools.

## Data availability statement

The data presented in the study are deposited in the National Center for Biotechnology Information repository, accession numbers are SRR21707708, SRR21707707, SRR21707706, SRR21707705, SRR21707704, SRR21707703.

## Author contributions

JiL, YY, and YZ contributed to conception and design of the study. JiL and YY organized the database. JiL wrote the first draft of the manuscript. YZ performed the statistical analysis. WL, MC, and HM wrote sections of the manuscript. AG and JuL contributed to funding acquisition, resources, supervision, and writing–review and editing. All authors contributed to the article and approved the submitted version.

## Funding

This work was supported by the grants from the Major Science and Technology Fund of Guangxi Province (AB21196020) and (AB20297006).

## Conflict of interest

The authors declare that the research was conducted in the absence of any commercial or financial relationships that could be construed as a potential conflict of interest.

## Publisher’s note

All claims expressed in this article are solely those of the authors and do not necessarily represent those of their affiliated organizations, or those of the publisher, the editors and the reviewers. Any product that may be evaluated in this article, or claim that may be made by its manufacturer, is not guaranteed or endorsed by the publisher.
